# Radiomics in distinguishing between lung adenocarcinoma and lung squamous cell carcinoma: a systematic review and meta-analysis

**DOI:** 10.3389/fonc.2024.1381217

**Published:** 2024-09-24

**Authors:** Lili Shi, Jinli Zhao, Zhichao Wei, Huiqun Wu, Meihong Sheng

**Affiliations:** ^1^ Medical School, Nantong University, Nantong, China; ^2^ Department of Radiology, Affiliated Hospital of Nantong University, Nantong, China; ^3^ Department of Radiology, The Second Affiliated Hospital of Nantong University and Nantong First People’s Hospital, Nantong, China

**Keywords:** radiomics, lung adenocarcinoma, lung squamous cell carcinoma, texture analysis, computed tomography, positron emission tomography, magnetic resonance imaging

## Abstract

**Objectives:**

The aim of this study was to systematically review the studies on radiomics models in distinguishing between lung adenocarcinoma (LUAD) and lung squamous cell carcinoma (LUSC) and evaluate the classification performance of radiomics models using images from various imaging techniques.

**Materials and methods:**

PubMed, Embase and Web of Science Core Collection were utilized to search for radiomics studies that differentiate between LUAD and LUSC. The assessment of the quality of studies included utilized the improved Quality Assessment of Diagnostic Accuracy Studies (QUADAS-2) and Radiomics Quality Score (RQS). Meta-analysis was conducted to assess the classification performance of radiomics models using various imaging techniques.

**Results:**

The qualitative analysis included 40 studies, while the quantitative synthesis included 21 studies. Median RQS for 40 studies was 12 (range -5~19). Sixteen studies were deemed to have a low risk of bias and low concerns regarding applicability. The radiomics model based on CT images had a pooled sensitivity of 0.78 (95%CI: 0.71~0.83), specificity of 0.85 (95%CI:0.73~0.92), and the area under summary receiver operating characteristic curve (SROC-AUC) of 0.86 (95%CI:0.82~0.89). As for PET images, the pooled sensitivity was 0.80 (95%CI: 0.61~0.91), specificity was 0.77 (95%CI: 0.60~0.88), and the SROC-AUC was 0.85 (95%CI: 0.82~0.88). PET/CT images had a pooled sensitivity of 0.87 (95%CI: 0.72~0.94), specificity of 0.88 (95%CI: 0.80~0.93), and an SROC-AUC of 0.93 (95%CI: 0.91~0.95). MRI images had a pooled sensitivity of 0.73 (95%CI: 0.61~0.82), specificity of 0.80 (95%CI: 0.65~0.90), and an SROC-AUC of 0.79 (95%CI: 0.75~0.82).

**Conclusion:**

Radiomics models demonstrate potential in distinguishing between LUAD and LUSC. Nevertheless, it is crucial to conduct a well-designed and powered prospective radiomics studies to establish their credibility in clinical application.

**Systematic review registration:**

https://www.crd.york.ac.uk/PROSPERO/display_record.php?RecordID=412851, identifier CRD42023412851.

## Introduction

According to the GLOBOCAN estimates of cancer incidence and mortality, lung cancer is the second most common cancer and has the highest mortality rate among all types of cancer (1). Non-small cell lung cancer (NSCLC) is the predominant subtype, making up approximately 85% of all lung cancer cases (2). Approximately 80% of NSCLC are attributed to lung adenocarcinoma (LUAD) and lung squamous cell carcinoma (LUSC), which are the primary pathological subtypes (3). The variations in histological and biological features between LUAD and LUSC lead to notable distinctions in their treatment plan, prognosis, and rates of relapse (4–6). For example, targeted therapy is more beneficial for LUAD, while LUSC is more susceptible to chemotherapy (2). Precise identification of pathological types of NSCLC can help clinicians take appropriate treatment in time to improve clinical outcomes. The gold standards for classifying LUAD and LUSC are still pathological diagnosis made from biopsy or surgical resection lesions. However, this method is invasive, may be accompanied by potential complications, and is not appropriate to perform in any cases. Additionally, it may not always be feasible to ascertain the histological subtype of small biopsies or cytology specimens obtained during diagnostic procedures. There is an urgent need for a precise and non-intrusive categorization of NSCLC prior to treatment.

Computed tomography (CT) is often the first choice of modality for the diagnosis of lung cancer. The patients with possible lung cancer are then offered positron emission tomography/computed tomography (PEC/CT) for staging. Magnetic resonance imaging (MRI) has aroused interest in lung cancer diagnosis due to its ionizing radiation-free, superior soft-tissue contrast, and unique morpho-functional imaging capacities (7). Sometimes, the diagnosis of lung cancer involves a combination of CT, PET/CT, and MRI (8). However, it poses a significant difficulty for medical professionals to visually anticipate the histological classification of NSCLC solely based on images, regardless of the modality, let alone to predict eligibility for personalized treatments, e.g. targeted therapies, and individual outcome. Radiomics, also known as a virtual biopsy, utilizes an extensive range of imaging characteristics to measure phenotypical variances from medical images and reveal additional concealed information in contrast to regular features (9). The radiomics method has been used for differential diagnosis, prognosis prediction, and treatment outcome prediction (10–12). Radiomics features can also be correlated with genetic mutations or alterations to help personalize the management of diseases (13).

The aim of this study was to examine the studies utilizing radiomics to differentiate between LUAD and LUSC and evaluate the performance of radiomics models in classifying histologic subtypes using images from various imaging techniques.

## Materials and methods

The study protocol was registered on the Prospective Register of Systematic Reviews with the registration number CRD42023412851. This systematic review and meta-analysis were conducted according to the PRISMA guidelines (14). The PRISMA checklist is shown in **Supplementary Table S1**.

### Search strategy

PubMed, Embase and Web of Science Core Collection were queried to identify relevant studies published until July 17, 2023, utilizing terms such as radiomics, lung adenocarcinoma, lung squamous cell cancer with Boolean logic operation. The search details were listed in **Supplementary Table S2**. The reference lists of included studies and relevant reviews were manually examined to identify any potential studies that may have been overlooked. There was no language limit.

### Study selection

We included studies that met the following criteria: (1) radiomics-based model for LUAD and LUSC classification; (2) radiomics features extracted from pre-treatment lung imaging irrespective of the modality of imaging; (3) patients were pathologically confirmed as LUAD or LUSC.

We excluded (1) articles that were not original full-text, such as reviews, letters, or commentaries, as well as conference abstract; (2) studies that did not provide information on LUAD and LUSC classification; (3) studies that used features other than radiomics to differentiate between LUAD and LUSC; (4) studies that used a sample that had already been used in another study; (5) studies that lacked sufficient information to assess their quality.

Two reviewers (LS, with 6 years of experience in chest image analysis, AND JZ, a broad-certified radiologist with 19 years of experience) individually examined tiles, abstracts, and assessed the full texts to determine eligibility. The disagreements were resolved by consensus.

### Date extraction

Basic information of each study including the surname of the first author and the publication year were extracted. The study’s characteristics such as sample size and study design were provided. The index test information consisted of the imaging modality used, segmentation method, software used for segmentation and radiomics feature extraction, the number of radiomics features extracted, feature selection method, classification model, and any non-radiomics features included in the model. Furthermore, the outcomes encompassed true positive, false positive, false negative and true negative, along with any other statistical data that might be used for calculation. If multiple classification objectives were presented in a study, only the information differentiating LUAD from LUSC based on radiomics features was extracted. If a study where multiple radiomics models were mentioned, the model with the highest area under the curve (AUC) was selected.

Data extraction was implemented independently by two reviewers (LS, ZW with 4 years of experience in chest image analysis). The disagreements were resolved by discussion.

### Quality assessment

The quality of the included studies was evaluated using the Improved Quality Assessment of Diagnostic Accuracy Studies (QUADAS-2) and Radiomics Quality Score (RQS). The purpose of QUADAS-2 is to evaluate the quality of primary diagnostic studies, which includes 4 key domains: patient selection, index test, reference standard, and flow and timing (15). The results of QUADAS-2 were recorded using Revman 5.4. The signaling questions of the 4 key domains were modified to tailor to our study (16). If all signaling questions of a domain were answered “yes”, the domain was considered at low risk of bias.

RQS, consisting of 16 items, was suggested as a means to enhance the radiomics workflow and has been extensively employed in evaluating the methodological quality of radiomics studies in systematic review (9). After evaluating the studies based on each item, a total score will be calculated for each study and displayed on a scale ranging from -8 to 36, which can be converted into a percentage. Scores below 0 will be considered as 0, while a score of 36 will be equivalent to 100%.

The quality of the included studies was evaluated by two separate reviewers (LS and JZ). Quality discrepancies were resolved through reassessment and discussion.

### Statistical analysis

The agreement between two reviewers on each item of RQS and each signaling question of QUADAS-2 was by expressed by a modified Fleiss kappa statistic (17). The inter-rater agreement of total RQS was measured using the interclass correlation coefficient (ICC) (18). R (version 4.2.2) was utilized for the computation. A significance level of less than 0.05 was deemed statistically significant.

Studies were pooled to estimate sensitivity and specificity along with 95% confidence intervals (CIs) using random-effect model. To reflect the synthesized diagnostic accuracy, the summary receiver operating characteristic (SROC) curve was constructed and the AUC was calculated. The chi-squared test was utilized to analyze the statistical heterogeneity among studies and the results were presented as the *I* ^2^ statistic. Significant heterogeneity was observed when *P*<0.1 and *I* ^2^>50%. Subgroup analysis was performed to detect the cause of heterogeneity. To evaluate the model’s stability, sensitivity analysis was conducted by plotting measures of goodness-of-fit, bivariate normality, influence analysis and outlier detection. An investigation of publication bias was conducted using Deeks’ funnel plot, and a *P*-value was computed using Deeks’ asymmetry test. Two-tailed *P*<0.05 was considered statistically significant.

## Results

### Study selection

**Figure 1:**
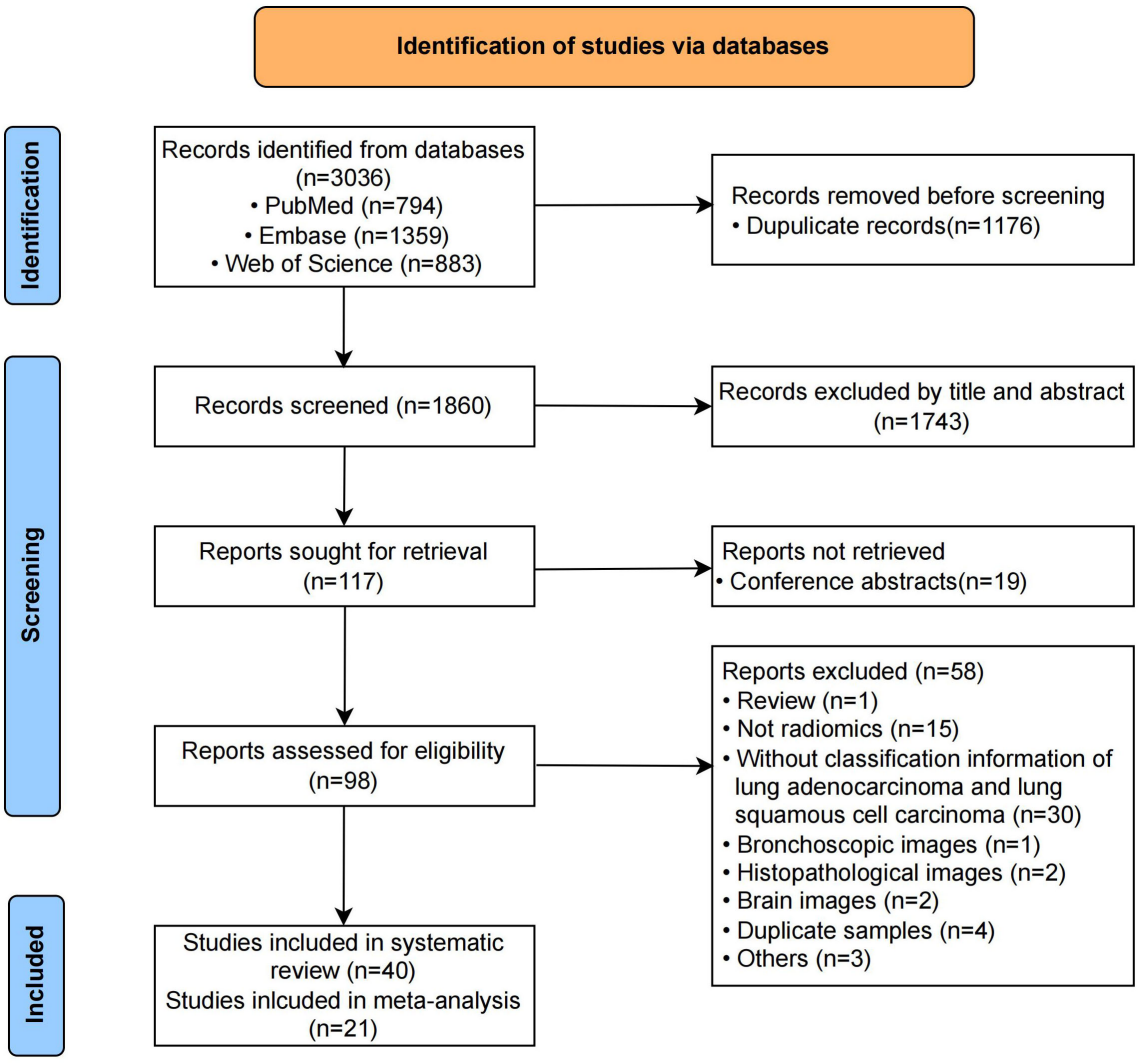
PRISMA flowchart of study selection.

The initial search and duplicate removal yielded 1860 unique records. After reading titles and abstracts, 1743 were eliminated. The remaining 117 full-texts were screened for eligibility. The number of records and reasons for removing are listed in **Figure 1**. Finally, 40 records were included in this systematic review, out of which 21 contained enough data to generate the diagnostic confusion matrix and were subsequently included in the meta-analysis. Neither their reference lists nor relevant reviews provided additional eligible studies.

### Study characteristics

The characteristics of studies included in this review were listed in **Table 1** (19–58). Studies were published between 2011 and 2023. All but one of the included studies enrolled patients retrospectively. The range of patients in the studies that were included varied from 30 to 1419. The imaging modalities used included CT, PET/CT, MRI and PET/MRI. Two studies (51, 58) constructed radiomics models based on images from two or more imaging modalities. Twenty-five studies segmented regions of interests manually, 4 automatically, and 11 semi-automatically. Most studies segmented the regions of tumors only, while two studies (35, 41) also segmented the peritumoral regions of interests. ITK-SNAP (8/40) was the most used segmentation software, next the LIFEx (3/40). Matlab (8/40) was used frequently to extract radiomics features, followed by pyradiomics (7/40). The extracted features included first-, second-, and higher-order features, along with shape features. Logistic regression (11/40) and SVM (10/40) were the most two commonly used classifiers. Sixteen studies included non-radiomics features in multivariable models such as gender, tumor location, and smoking status.

**Table 1:** Characteristic of included studies.

Study ID	Patients (LUAD)	Imaging modality	Study design	Segmentation method	Segmentation software	Radiomics feature extraction algorithm or software	No. radiomics features extracted	Radiomics features	Radiomics feature selection methods	Classification model	Non-radiomics features in model
Basu 2011 (19)	74 (38)	CT	R	A	Lung Tumor Analysis software suite	Lung Tumor Analysis software suite	317	Texture, morphological, geometric, intensity	–	J48	–
Wu 2016 (20)	350 (214)	CT	R	M	–	Matlab	440	Intensity, shape, texture	Correlation analysis, univariate analysis, ReliefF	Naive Bayesian	–
Yu 2017 (21)	434(324)	CT	R	A	Toboggan Based Growing Automatic Segmentation	–	52	First-, second-, high-order	Iterative forward including and backward elimination	RUSBoost	–
Haga 2018 (22)	40(21)	CT	R	S	Pinnacle3 v9.10	Matlab	476	Shape, global, GLCM, GLRLM, GLSZM, NGTDM, wavelet filter	Random permutation test, interobserver variation analysis, correlation analysis	Naive Bayesian	–
Sandino 2018 (23)	40(20)	CT	R	M	–	–	–	Texture	–	SVM	–
Tsubakimoto 2018 (24)	43(25)	CT	P	S	Synapse Vincent	JMP Pro	–	Histogram	–	LR	SUVmax(RG)
Zhu 2018 (25)	129(76)	CT	R	M	ITK-SNAP	Matlab	485	Intensity, shape and size, texture, and wavelet filter	LASSO	LASSO	–
Bashir 2019 (26)	206(129)	CT	R	M	ITK-SNAP	Matlab	756	Geometric, first-, second-, higher-order, model-based, wavelet filter	Pairwise correlation	RF	Air bronchogram, ground-glass component, cavitation
Digumarthy 2019 (27)	94(69)	CT	R	S	TexRAD	CTTA software	11		Pearson correlation analysis	LR	Smoking, longest diameter, longest perpendicular diameter
E 2019 (28)	181(88)	CT	R	S	–	Matlab	1695	Shape, size, boundary sharpness, texture	CCC, hierarchical clustering, mRMR, incremental forward search	SVM	–
Liu 2019 (29)	87(47)	CT	R	M	Digital biopsy	MaZda	261	Histogram, GLCM, RLM, gradient, AR	Fisher Score	SVM	Location
Yamada 2019 (30)	170(82)	CT	R	M	–	–	486	Histogram, texture, wavelet fliter	LASSO	SVM	–
Alvarez-Jimenez 2020 (31)	101(49)	CT	R	M	–	–	120	Haralick texture, statastics	Spearman’s rank correlation	SVM	–
Brunese 2020 (32)	130(98)	CT	R	M	–	–	14	shape	–	Neural Net	–
Han 2020 (33)	70(41)	CT	R	M	CTKinetics APP	CTKinetics APP	42	Histogram, GLCM, GLRLM	t-test, wilcox test, spearman correlation analysis	LR	–
Tomori 2020 (34)	40(22)	CT	R	M	LIFEx	LIFEx	42	Histogram, shape, GLCM, GLRLM, NGLDM, GLZLM	Mann-Whitney U test	LR	SUVmax
Vuong 2020 (35)	105(63)	CT	R	M	MIM VISTA	Z-Rad	154	Intensity, texture	ICC, PCA, univariate logistic regression	LR	–
Chanuzwa 2021 (36)	272(185)	CT	R	A	–	CNN	–	–	–	CNN	–
Li 2021 (37)	121(55)	CECT	R	M	ITK-SNAP	PyRadiomics	107	First-order, shape, GLDM, GLSZM, GLRLM, GLCM, NGTDM	LASSO	FNN	–
Liu 2021 (38)	126(72)	CT	R	S	ITK-SNAP	Pyradiomics	107	Shape, first-order, GLDM, GLRLM, GLSZM, GLCM, NGTDM	Fisher score	RF	–
Marentakis 2021 (39)	102(48)	CT	R	M	–	–	529	First-order, shape, texture, wavelet filter	–	KNN	–
Chen 2022 (40)	129(87)	CT	R	S	ITK-SNAP	A.K. Software	107	First-order, shape, texture	ICC, LASSO	LR	Gender, distant metastasis, NIC_VP_
Tang 2022 (1) (41)	105(58)	CT	R	M	Custom-developed package	PyRadiomics	1023	First-, higher-order, GLCM, GLRLM, NGTDM, GLSZM, GLDM	t-test, SVM-RFE	Ensemble classifier	–
Chen 2023 (42)	324(157)	CT	R	M	–	PyRadiomics	1158	First-order, shape, texture, transform-based	L_2,1-norm_ minimization	SVM	–
Song 2023 (43)	868(600)	CT	R	M	ITK-SNAP	PyRadiomics	1409	Shape, intensity, texture, higher-order	L _2,1-norm_ minimization	Bagging-AdaBoost-SVM	–
Ha 2014 (44)	30(17)	PET/CT	R	M	MaZda	MaZda	>200	Texture	Fisher coefficient, minimization of both classification error probability and average correlation, mutual information	LDA	–
Ma 2018 (45)	299(125)	PET/CT	R	M	–	Matlab	–	Texture	–	SVM	Color features
Hyun 2019 (46)	396(210)	PET/CT	R	M	–	LIFEx	40	First-order, GLCM, NGLDM, GLRLM, GLZLM	Gini coefficient	LR	Gender, SUVmax, total lesion glycolysis, age
Sha 2019 (47)	100(61)	PET/CT	R	M	MIM Maestro	Chang-Gung Image Texture Analysis	107	Texture, shape	LASSO	LR	Smoking
Ayyildiz 2020 (48)	93(39)	PET/CT	R	A	Random walk	Matlab	39	GLCM, GLRLM, GLSZM, NGTDM	CFS subset evaluator	Ensemble classifier	–
Han 2021 (49)	1419(867)	PET/CT	R	S	MATLAB	PyRadiomics	688	First-order, GLCM, GLRLM, GLSZM, GLDM, wavelet filter	L _2,1_ norm regularization	LDA	–
Ji 2021 (50)	416	PET/CT	R	S	Chang-Gung Image Texture Analysis	Chang-Gung Image Texture Analysis	54	Texture	LASSO	LR	Location
Ren 2021 (51)	315(193)	PET/CT	R	M	Inveon Research Workplace	Chang-Gung Image Texture Analysis	212	GLCM, GLRM, GLNIDM, GLSZM, TFC, TFCCM, NGLD	Univariate analysis, LASSO	LASSO	Gender, size, SCCA, CYFRA21.1
Shen 2021 (52)	250(150)	PET/CT	R	S	ITK-SNAP	PyRadiomics	385	First-order, shape, GLRLM, GLSZM, GLDM, GLCM, NGTDM, CoLIAGe features	Spearman correlation analysis, chi-square test, SVM-RFE	SVM-RBF	Gender
Zhou 2021 (53)	452(329)	PET/CT	R	S	LIFEx	LIFEx	48(PET) 41(CT)	First-, second-, higher order	GBDT	GBDT(PET) RF(CT)	–
Zhao 2022 (54)	120(62)	PET/CT	R	S	LIFEx	LIFEx	91	First-, second-order, conventional indices	ICC, Boruta algorithm	SVM	Gender, smoking, CEA, SCCA
Tang 2020 (55)	148(80)	MRI	R	M	Custom-developed package.	Matlab	1404	Histogram, CM, RLM, NGTDM, GLSZM	t-test, SVM-RFE	LR	Age, smoking, location, LD, LPD
Yang 2023 (56)	71(46)	MRI	R	M	3D Slicer	SlicerRadiomics	–	Shape, first-, second-order, wavelet filters	LASSO	LR	Smoking
Bebas 2021 (57)	44(24)	PET/MRI	R	M	–	QMazda	303	First-order, GLCM, GLRLM, AR, GMF, HOG, LBP, Gabor, wavelet	t-test, Mann-Whitney U test	SVM	–
Tang 2022 (2) (58)	80 (47)	PET/MRI, CT	R	M	ITK-SNAP	uAI Research Portal	2264	First-order, GLRLM, GLSZM, NGTDM, GLCM, GLDM	F test and LASSO	Gaussian process	Position, TLG, volume

LUAD, lung adenocarcinoma; R, retrospective; P, prospective; S, semi-automatic; M, manual; A, automatic; GLCM, gray level co-occurrence matrix; GLRLM, gray level run length matrix; GLSZM, gray level size zone matrix; NGTDM, neighboring gray tone difference matrix; NGLDM, neighborhood gray-level different matrix; GLZLM, gray-level zone length matrix; RLM, run-length matrix; NGLD, neighboring gray level dependence; TFC, texture feature coding; TFCCM, texture feature coding co-occurrence matrix; AR, autoregressive model; CM, co-occurrence matrices; GMF, gradient map features; LBP, local binary patterns; HOG, histogram of oriented gradients; LASSO, least absolute shrinkage and selection operator; CCC, concordance correlation coefficient; mRMR, max-relevance and min-redundancy; RFE, recursive feature elimination; ICC, intraclass correlation coefficient; PCA, principal component analysis; GBDT, gradient boosting decision tree; LDA, linear discriminant analysis; SVM, support vector machine; LR, logistic regression; RF, random forest; CNN, convolutional neural network; QDA, quadratic discriminant analysis; RBF, radial basis function; TLG, total lesion glycolysis; NIC, normalized iodine concentration; VP, venous phase; FNN, feedforward neural network; CEA, carcinoembryonic antigen; SCCA, squamous cell carcinoma antigen.

### Study quality

**Figure 2:**
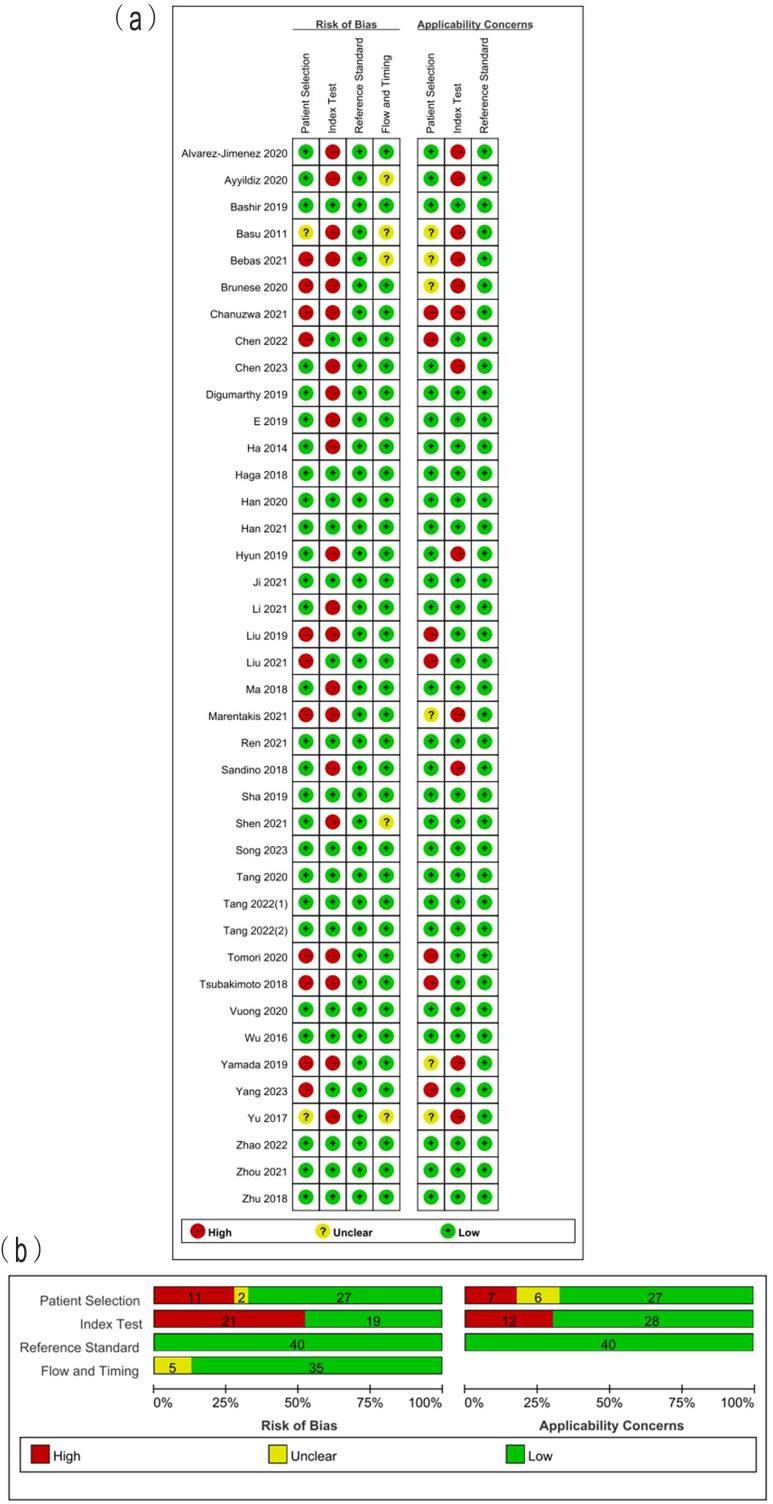
**(A)** Methodological quality assessment of individual studies and **(B)** summary of the methodological quality of included studies.

The summarized QUADAS-2 results are showed in **Figure 2**. In the domain of patient selection, 2 studies did not provide the information of patient, 5 did not randomly or consecutively enrolled patients, and the exclusion criteria in 7 studies were inappropriate. With regards to the domain of index test, 4 studies did not describe the imaging acquisition well, 6 did not describe the segmentation methods in detail, 6 did not describe the feature extraction software. Independent validation was missed in 17 studies. All included studies used the reference standard which could correctly classify between LUAD and LUSC. The interval between imaging and reference standards was not reported in 5 studies. The high concern of applicability was observed in the aspects of index test (12/40) and patient selection (7/40). The answers to 9 signaling question of included studies are showed in **Supplementary Table S3**. The absolute agreement of 9 signaling questions ranged from 86.3% to 100%.

The total RQS of included studies ranged from -5 to 19, with median of 12 (**Table 2**). The median RQS proportion was 33.3%, with a maximum of 52.8%. The RQS guideline did not define low or high quality clearly. However, 17 studies scored less than 30%. No studies included in the analysis performed multiple segmentation or cost-effective analysis. Feature reduction was conducted in 90% studies prior to modeling. More than half of the studies (23/40) used independent validation sets. Only 6 studies reported calibration statistics. Six studies reported the potential clinical utility. Twenty-nine studies published neither code nor data. The scores for each item and total scores of each study are presented in **Table 2**. The Fleiss kappa statics varied from 84.4% to 100% for each item and ICC of 98.1% (95%CI: 96.4%~99.0%) for the overall RQS showed a satisfactory level of agreement between the two reviewers, as shown in **Table 2**.

**Table 2:** RQS of included studies.

StudyID	Image protocol (0/1/2)	Multiple segmentations (0/1)	Inter-scanner differences (0/1)	Imaging multiple times (0/1)	Feature reduction (-3/3)	Non- radiomic feature (0/1)	Biological correlates (0/1)	Cut-offs (0/1)	Discrimination (0/1/2)	Calibration (0/1/2)	Prospective (0/7)	Validation (-5/2/3/4/5)	gold standard (0/2)	Clinical utility (0/2)	Cost (0/1)	Open science (0/1/2/3/4)	RQS (total) (-8~36)	RQS (%) (0~100%)
Basu 2011 (19)	0	1	0	0	-3	0	0	0	0	0	0	-5	2	0	0	0	-5	0
Wu 2016 (20)	1	1	0	0	3	0	0	1	2	0	0	3	2	0	0	3	16	44.4
Yu 2017 (21)	1	1	0	0	3	0	0	0	2	0	0	-5	2	0	0	0	4	11.1
Haga 2018 (22)	1	1	1	0	3	0	0	0	2	0	0	2	2	0	0	0	12	33.3
Sandino 2018 (23)	1	1	0	0	-3	0	0	0	2	0	0	-5	2	0	0	1	-1	0
Tsubakimoto 2018 (24)	1	0	1	0	3	1	1	1	2	0	7	-5	2	0	0	0	14	38.9
Zhu 2018 (25)	1	1	0	0	3	0	1	0	2	0	0	2	2	0	0	0	12	33.3
Bashir 2019 (26)	1	1	0	0	3	1	1	0	2	0	0	3	2	0	0	0	14	38.9
Digumarthy 2019 (27)	1	0	0	0	3	1	1	0	2	0	0	-5	2	0	0	0	5	13.9
E 2019 (28)	1	0	0	0	3	0	1	0	2	0	0	-5	2	0	0	0	4	11.1
Liu 2019 (29)	1	0	1	0	3	1	1	0	2	0	0	-5	2	0	0	0	6	16.7
Yamada 2019 (30)	1	1	0	0	3	0	0	0	2	0	0	-5	2	0	0	1	5	13.9
Alvarez-Jimenez 2020 (31)	1	1	0	0	3	0	0	0	2	0	0	-5	2	0	0	1	5	13.9
Brunese 2020 (32)	1	1	0	0	-3	0	0	0	2	0	0	-5	2	0	0	1	-1	0
Han 2020 (33)	1	0	1	0	3	0	1	1	2	0	0	2	2	0	0	0	13	36.1
Tomori 2020 (34)	1	1	1	0	3	1	1	1	2	0	0	-5	2	0	0	0	8	22.2
Vuong 2020 (35)	1	0	0	0	3	0	1	0	2	0	0	3	2	0	0	0	12	33.3
Chanuzwa 2021 (36)	0	1	0	0	-3	0	0	0	2	0	0	3	2	0	0	0	5	13.9
Li 2021 (37)	1	1	0	0	3	0	0	0	2	0	0	-5	2	0	0	0	4	11.1
Liu 2021 (38)	1	1	1	0	3	0	0	0	2	0	0	2	2	0	0	0	12	33.3
Marentakis 2021 (39)	1	1	0	0	3	0	0	0	2	0	0	2	2	0	0	1	12	33.3
Chen 2022 (40)	1	0	1	0	3	1	1	0	2	2	0	2	2	2	0	0	17	47.2
Tang 2022 (1) (41)	1	1	1	0	3	0	0	0	2	0	0	2	2	0	0	1	13	36.1
Chen 2023 (42)	1	1	0	0	3	0	0	0	2	0	0	2	2	0	0	1	12	33.3
Song 2023 (43)	1	1	0	0	3	0	1	0	2	0	0	4	2	0	0	1	15	41.7
Ha 2014 (44)	1	0	1	0	3	0	1	0	0	0	0	-5	2	0	0	1	4	11.1
Ma 2018 (45)	1	0	0	0	3	1	1	1	2	0	0	-5	2	0	0	0	6	16.7
Hyun 2019 (46)	1	0	1	0	3	1	1	0	2	0	0	2	2	0	0	0	13	36.1
Sha 2019 (47)	1	1	1	0	3	1	0	0	2	0	0	2	2	0	0	1	14	38.9
Ayyildiz 2020 (48)	0	0	1	0	3	0	0	0	2	0	0	-5	2	0	0	0	3	8.3
Han 2021 (49)	1	0	1	0	3	0	0	0	2	0	0	2	2	0	0	0	11	30.6
Ji 2021 (50)	1	0	1	0	3	1	1	0	2	2	0	2	2	2	0	0	17	47.2
Ren 2021 (51)	1	1	1	0	3	1	1	0	2	1	0	2	2	2	0	0	17	47.2
Shen 2021 (52)	1	0	1	0	3	1	1	0	2	0	0	-5	2	0	0	0	6	16.7
Zhou 2021 (53)	1	0	1	0	3	0	0	0	2	0	0	2	2	0	0	0	11	30.6
Zhao 2022 (54)	1	1	1	0	3	1	0	0	2	0	0	2	2	0	0	0	13	36.1
Tang 2020 (55)	1	1	1	0	3	1	1	1	2	2	0	2	2	2	0	0	19	52.8
Yang 2023 (56)	1	1	0	0	3	1	1	0	2	1	0	2	2	2	0	0	16	44.4
Bebas 2021 (57)	0	0	0	0	3	0	0	0	0	0	0	-5	2	0	0	0	0	0
Tang 2022 (2) (58)	1	1	1	0	3	1	1	0	2	1	0	2	2	2	0	0	17	47.2
Inter-rater agreement (%)	87.5	84.8	95.0	100.0	100.0	100.0	95.0	100.0	100.0	90.6	100.0	93.1	100.0	100.0	100.0	84.4	98.1	98.0

### Diagnostic efficacy

**Figure 3:**
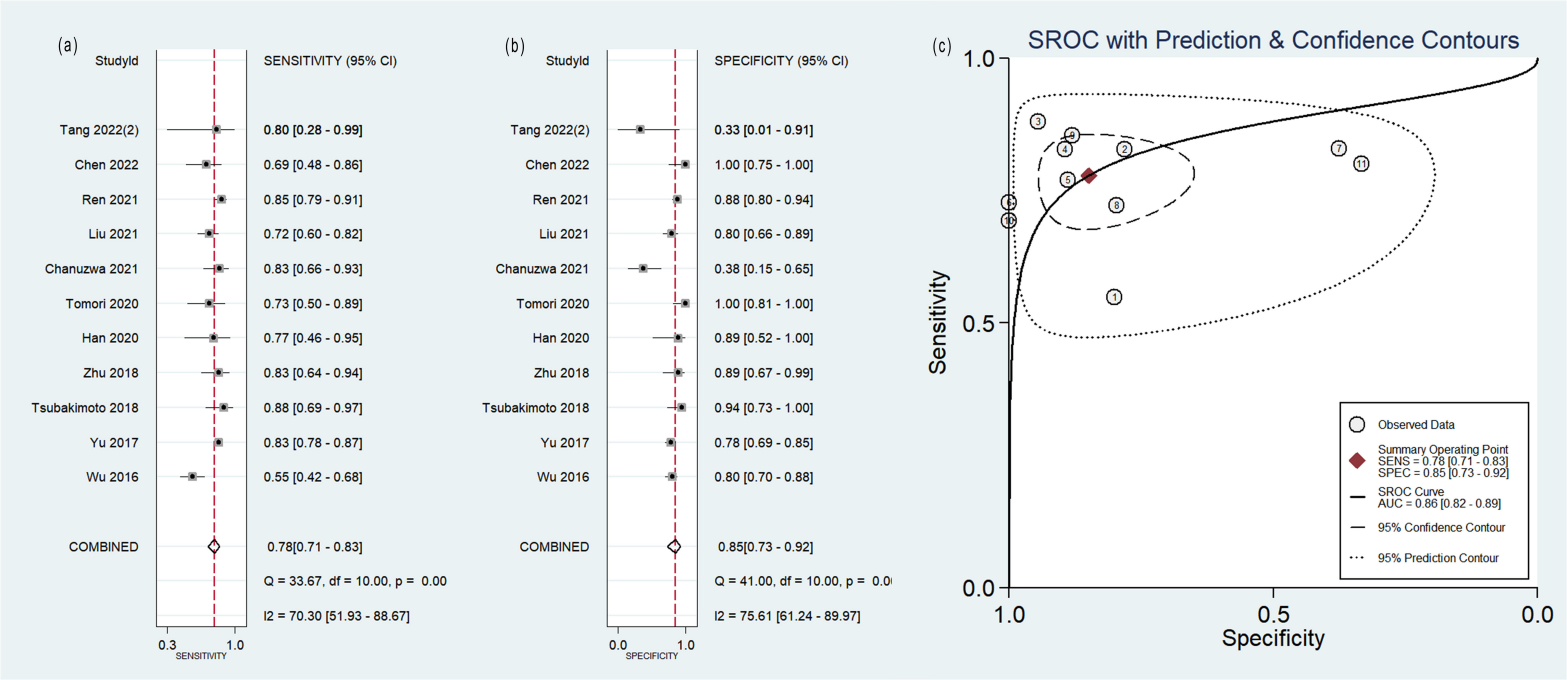
Forest plot of the pooled **(A)** sensitivity, **(B)** specificity, and **(C)** SROC curve for radiomics model based on CT images in distinguishing between lung adenocarcinoma and lung squamous cell carcinoma.

**Figure 4:**
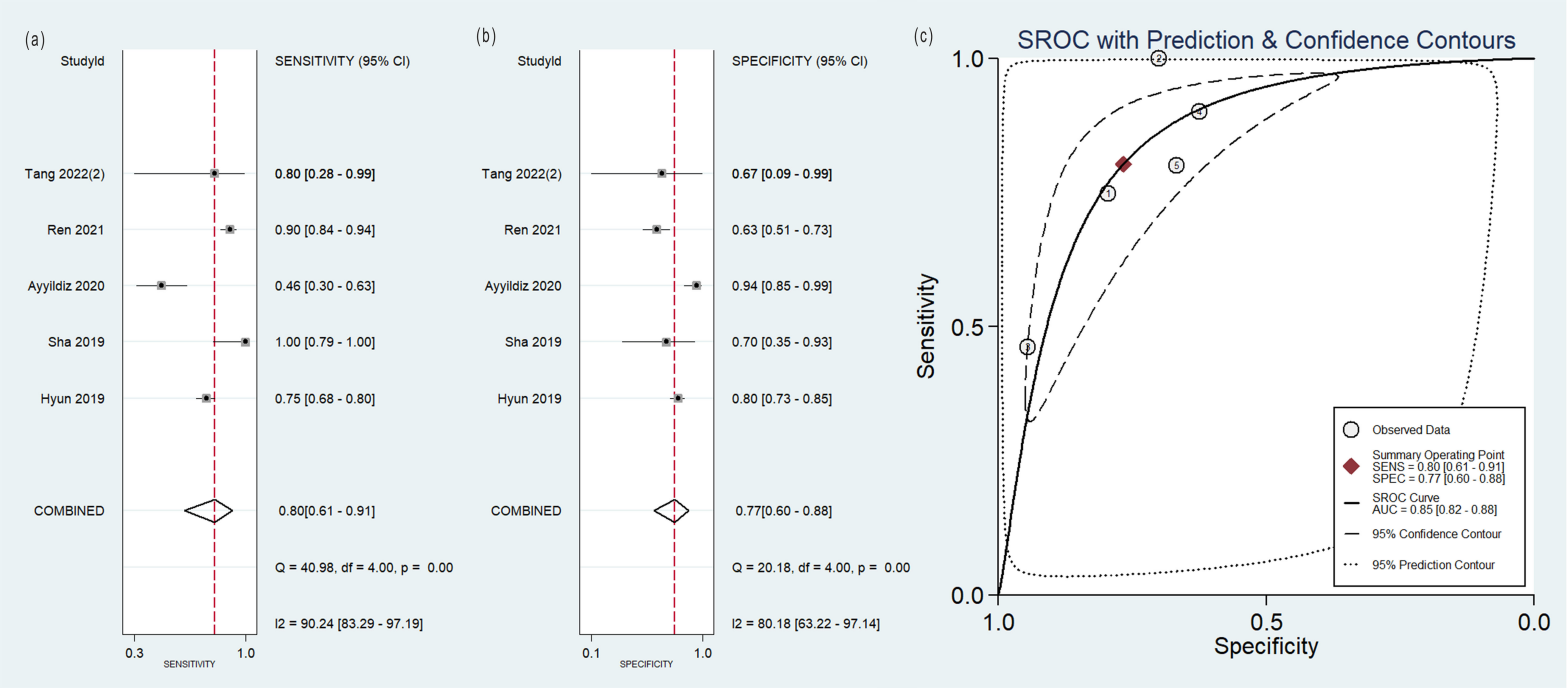
Forest plot of the pooled **(A)** sensitivity, **(B)** specificity, and **(C)** SROC curve for radiomics model based on PET images in distinguishing between lung adenocarcinoma and lung squamous cell carcinoma.

**Figure 5:**
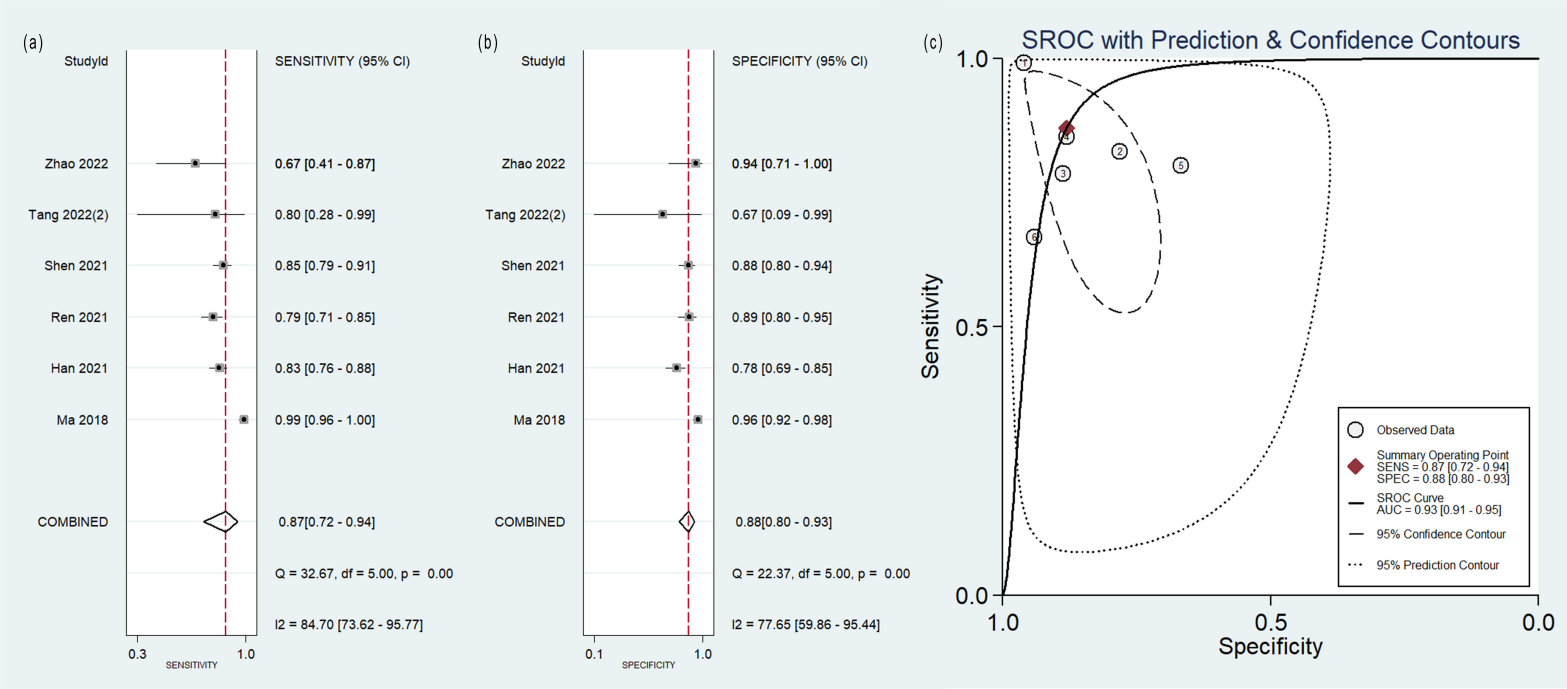
Forest plot of the pooled **(A)** sensitivity, **(B)** specificity, and **(C)** SROC curve for radiomics model based on PET-CT images in distinguishing between lung adenocarcinoma and lung squamous cell carcinoma.

**Figure 6:**
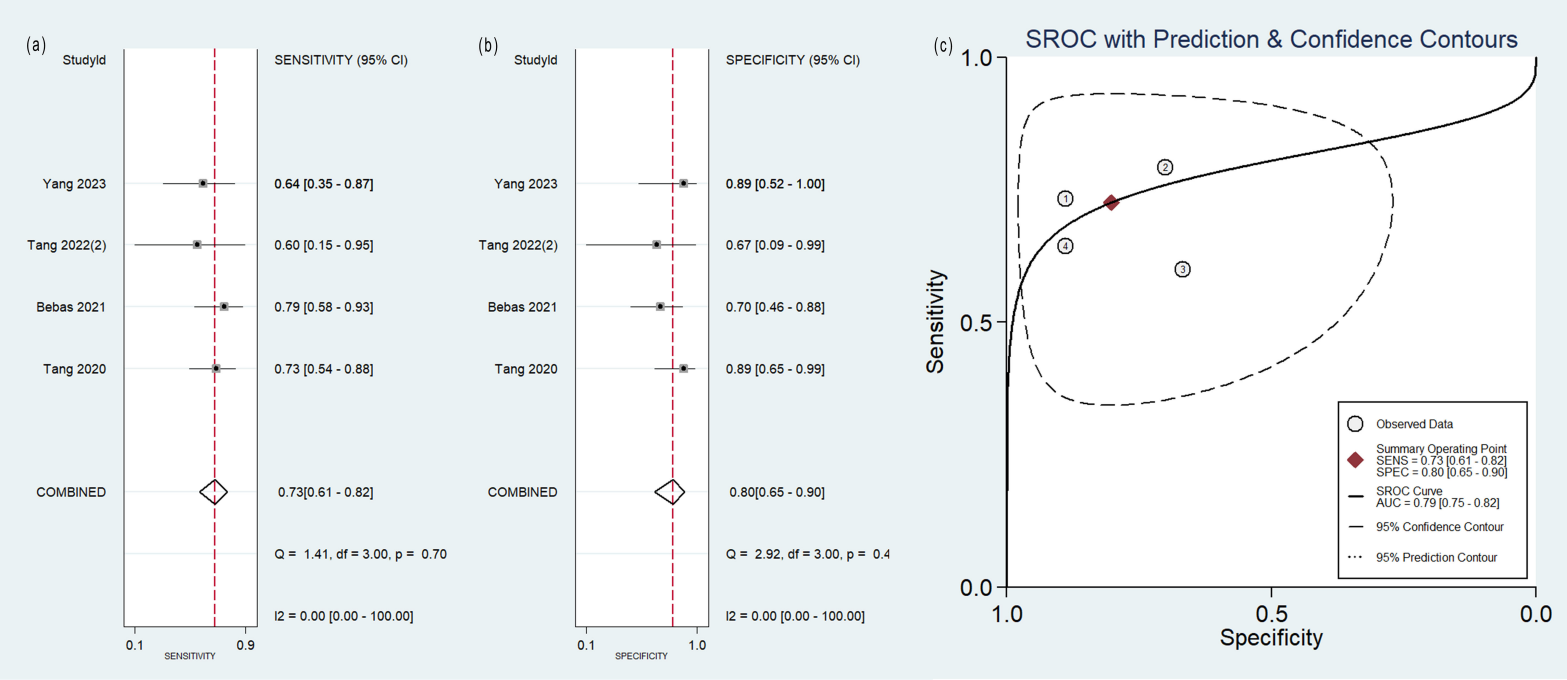
Forest plot of the pooled **(A)** sensitivity, **(B)** specificity, and **(C)** SROC curve for radiomics model based on MRI images in distinguishing between lung adenocarcinoma and lung squamous cell carcinoma.

The pooled analysis was performed according to radiomics studies based on various imaging techniques (**Supplementary Table S4**). Analysis could not be performed on only one study that utilized PET/MRI images. **Table 3** and **Figures 3**–**6** presented the combined sensitivity, specificity, and SROC-AUC for CT images, PET images, PET-CT images and MRI images. The radiomics model utilizing PET-CT images exhibited the greatest combined effects magnitudes, with a sensitivity of 0.87(95%CI: 0.72~0.94), specificity of 0.88(95%CI: 0.80~0.93) and SROC-AUC of 0.93(95%CI: 0.91~0.95), correspondingly. The radiomics model, which utilized CT and PET images, demonstrated favorable diagnostic performance with an SROC-AUC of 0.86 (95%CI: 0.82~0.89) and 0.85(95%CI: 0.82~0.88). Additionally, it exhibited moderate performance when applied to MRI images, achieving an SROC-AUC of 0.79 (95%CI: 0.75~0.82).

**Table 3:** The meta-analysis results.

Imaging modality	No. of study	Sensitivity (95%CI)	Specificity (95%CI)	SROC-AUC (95%CI)
CT	11	0.78(0.71~0.83)	0.85(0.73~0.92)	0.86(0.82~0.89)
	5 (With non-radiomics features)	0.82(0.73~0.88)	0.95(0.67~0.99)	0.89(0.86~0.91)
	6 (Without non-radiomics features)	0.76(0.66~0.84)	0.77(0.66~0.85)	0.83(0.80~0.86)
	9(Two outlier studies excluded)	0.81(0.75~0.85)	0.87(0.78~0.93)	0.87(0.84~0.90)
PET	5	0.80(0.61~0.91)	0.77(0.60~0.88)	0.85(0.82~0.88)
PET/CT	6	0.87(0.72~0.94)	0.88(0.80~0.93)	0.93(0.91~0.95)
	5(One outlier study excluded)	0.82(0.78~0.85)	0.86(0.79~0.91)	0.86(0.83~0.89)
MRI	4	0.73(0.61~0.82)	0.80(0.65~0.90)	0.79(0.75~0.82)
PET/MRI	1	0.80	0.67	

No heterogeneity was observed among the 4 studies that utilized MRI radiomics features, as indicated by *I^2^* value of 0% for the combined sensitivity and specificity (**Figure 6**). With regard to the pooled analysis based on other imaging modalities, there was substantial heterogeneity among studies with *I^2^* >50% (**Figures 3**-**5**).

Out of the 11 studies that developed radiomics models using CT images, 5 studies incorporated non-radiomics features in their models, resulting in higher sensitivity (0.82 compared to 0.76), specificity (0.95 compared to 0.77) and SROC-AUC (0.89 compared to 0.83) (**Table 3**, **Supplementary Figure S1**) than the 6 studies did not include non-radiomics features (**Table 3**, **Supplementary Figure S2**).

### Sensitivity analysis

**Figure 7:**
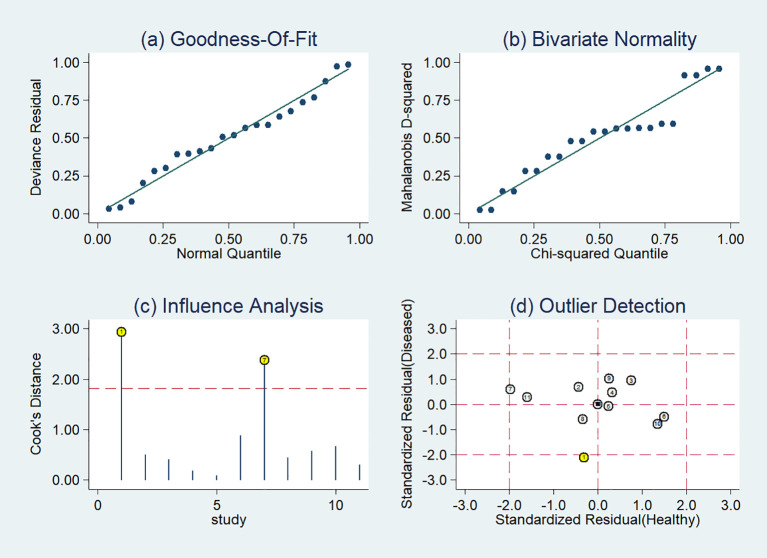
Sensitivity analysis of the included studies developing radiomics models in distinguishing between lung adenocarcinoma and lung squamous cell carcinoma utilizing CT images: **(A)** goodness of fit, **(B)** bivariate normality, **(C)** influence analysis, **(D)** outlier detection.

The sensitivity analysis showed two studies had impact on the pooled results of radiomics studies utilizing CT images (**Figure 7**). After removing the two studies (20, 36), the combined sensitivity, specificity, and SROC-AUC were 0.81(95%CI: 0.75~0.85), 0.87(95%CI: 0.78~0.93), and 0.87(95%CI: 0.84~0.90), correspondingly (**Table 3**, **Supplementary Figure S3**).

**Figure 8:**
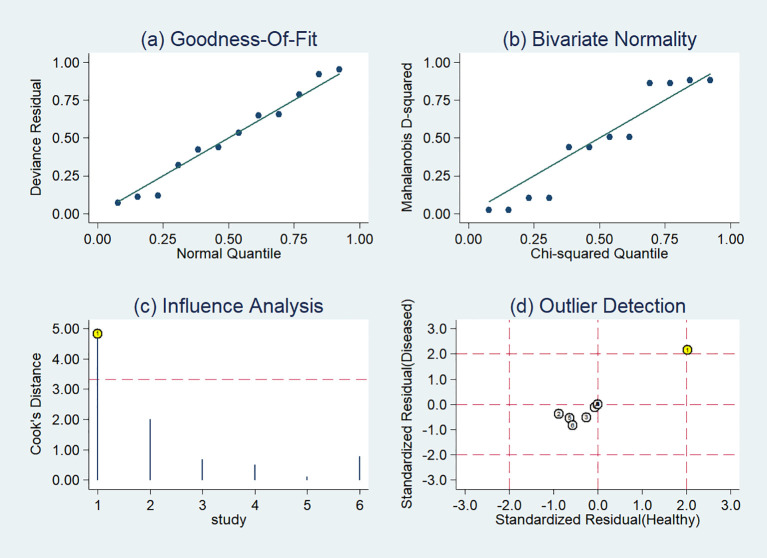
Sensitivity analysis of the included studies developing radiomics models in distinguishing between lung adenocarcinoma and lung squamous cell carcinoma utilizing PET-CT images: **(A)** goodness of fit, **(B)** bivariate normality, **(C)** influence analysis, **(D)** outlier detection.

The sensitivity analysis detected one outlier study (45) with the highest diagnostic performance (sensitivity of 0.99 and specificity of 0.96) among the included studies based on PET-CT images (**Figure 8**). By omitting the study, the combined sensitivity, specificity, and SROC-AUC were 0.82(95%CI: 0.78~0.85), 0.86(95%CI: 0.79~0.91) and 0.86(95%CI: 0.83~0.89), respectively (**Table 3**, **Supplementary Figure S4**). The study also influenced the heterogeneity. When it was removed, the heterogeneity indicator *I^2^* of sensitivity, and specificity, decreased from 84.7% to 21.2%, 77.6% to 46.1%, respectively.

The sensitivity analysis of pooled analysis based on PET images (**Supplementary Figure S5**) and MRI images (**Supplementary Figure S6**) did not find any studies impacting the pooled results.

### Publication bias

**Figure 9:**
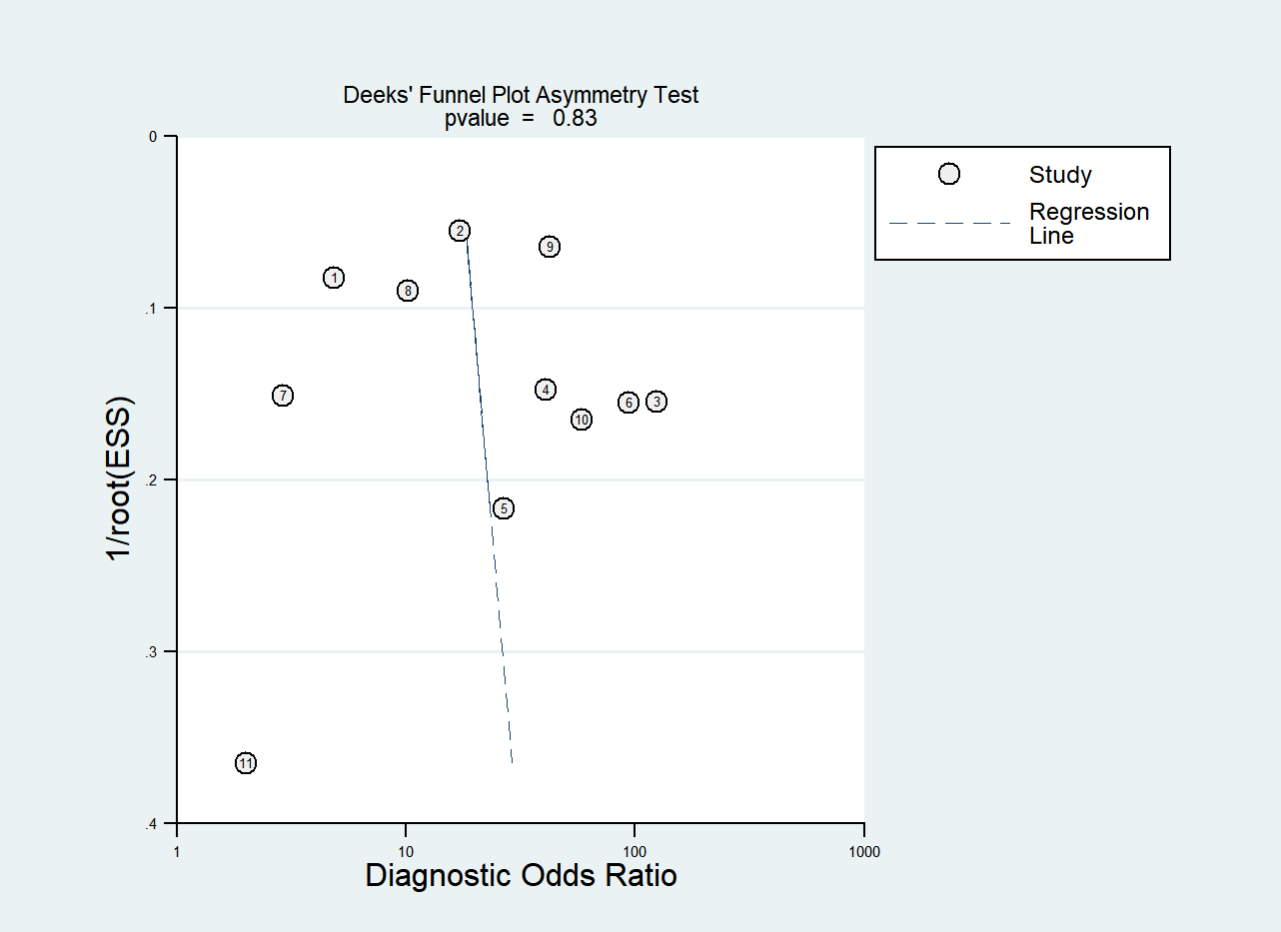
Deek’s funnel plot for radiomics studies based on CT images in distinguishing between lung adenocarcinoma and lung squamous cell carcinoma.

**Figure 9** displays Deeks’ funnel plot and the result of Deeks’ asymmetry test, which showcases the publication bias of the studies that developed radiomics classification models based on CT images. The figure did not exhibit evident asymmetry with *P* value of 0.83. The assessment of publication bias was not conducted for other subgroups due to the limited number of studies, which could result in an inconclusive funnel plot (59).

## Discussion

Radiomics has the potential to offer noninvasive diagnostic data on lesions using medical images enhancing the early identification of lung cancer histological subtype in certain patients who are ineligible for biopsy or surgical procedures. The meta-analysis findings indicated that the radiomics approach proved to be effective in the classification of LUAD and LUSC.

Radiomics has been used for more than a decade, but the clinical application suffers from numerous limitations. RQS was proposed to establish a standardized guideline for radiomics in 2017. The overall quality of studies included in this systematic review was undesirable. All but only one study was retrospectively designed which limited the generalizability of the classification model. The lack of code and data hindered the ability to replicate findings in future studies. Discrimination and calibration are the most commonly used metrics when evaluating the predictive models. Nevertheless, the calibration metric was disregarded in 85% the studies that were included. The percentage of RQS of 17 studies was lower than 30%, mainly due to the lack of model validation, whether internally or externally. Cost-effectiveness is a vital factor in incorporating a technique into everyday clinical practice (60), but the cost-effectiveness analyses were missed in all included studies.

The results of quality assessment according to improved QUADAS-2 showed that the high risk of bias was found in patient selection and index test, while the unclear risk of bias was found in flow and timing. For instance, some studies (29, 38, 40) excluded the small lesions for texture analysis, leading to the high risk of bias in the domain of patient selection. As a result of the growing utilization of lung cancer screenings, there is a higher probability of early detection for small lesions. Sixteen studies were deemed to have a low risk of bias in the four domain and low concerns regarding applicability. These studies also exhibited the percentage of RQS exceeding 30%. The agreement analysis findings for RQS and QUADAS-2 demonstrated the dependability of the quality assessment outcomes of the included studies.

CT is the most commonly used examination of the chest. In the present study, 65% (26/40) radiomics studies were based on CT images and 27.5% (11/40) with sufficient data were included in the meta-analysis. The results of the sensitivity analysis indicated the robustness of the diagnostic performance of radiomics model based on CT images, with sensitivity ranging from 0.81 to 0.78, specificity from 0.87 to 0.85, and SROC-AUC from 0.87 to 0.86, after the removal of two outlier studies. In subgroup analysis, the radiomics models incorporating non-radiomics features exhibited superior performance compared to those that did not include them. Non-radiomics features such as clinical, genetic and metabolic data can assist the histological classification of the lesions. However, the heterogeneity remained significant. Non-radiomics features could not explain the heterogeneity.

The pooled diagnostic effect sizes of radiomics models were the best based on PET-CT images. That PET-CT modality provided the anatomical and metabolic information of the tumors might be the reason. When the outlier study was omitted, the SROC-AUC of models based on PET-CT images was decreased from 0.93 to 0.86, which was equivalent to that of CT. Meanwhile, the heterogeneity among studies decreased significantly, which indicated the omitted study might be one of the sources of heterogeneity. The model in the omitted study with the highest sensitivity and specificity included radiomics features and color features. While the classification model of other studies in this meta-analysis did not incorporate color features, previous studies indicated that color features, in addition to texture, could be a valuable image characteristic (61, 62).

The models relying on MRI images exhibited lower classification performance compared to other models, achieving an SROC-AUC of 0.79. MRI is not a routine examination for lung cancer. Compared with CT, MRI has poorer spatial resolution, requires longer examination time and is more expensive. Nevertheless, MRI outperforms CT in cases where the lesion is located at the center or at the base/apex of the lung (56).

Despite being the initial endeavor to examine the classification performance of radiomics in distinguishing LUAD and LUSC through a systematic review of previous studies, there are still some limitations. Firstly, insufficient studies based on PET images, PET-CT images, and MRI images were included in the meta-analysis, which hindered the exploration of heterogeneity through meta-regression and subgroup analysis. In the radiomics studies based on CT images, the subgroup analysis was conducted. However, the grouped sample size might be insufficient to perform additional subgroup analyses, such as modeling method and segmentation method. Second, the heterogeneity of studies incorporated in the quantitative synthesis could arise from several aspects, including the types of scanner machines, segmentation techniques, radiomics feature extraction methods, and modeling methods. The presence of heterogeneity might decrease the dependability of our findings. As the number of studies increase, scientific data aggregation will be possible in the future. Third, other tools such as Checklist for Artificial intelligence in Medical Imaging (CLAIM) (63) and Prediction model Risk Of Bias ASsessment Tool (PROBAST) (64) can also be utilized to investigate the methodologic quality of the studies included. RQS and QUADAS-2 have limitations. Still, they are more suitable for the methodologic assessment of radiomics studies. In turn, the quality of radiomics studies can be improved if these methodological assessment tools are taken into account at the stage of study design.

To sum up, radiomics models hold potential for distinguishing between LUAD and LUSC. Nevertheless, it is crucial to conduct well-designed and powered prospective radiomics studies in order to establish their credibility in clinical application.

## Data Availability

The datasets presented in this study can be found in online repositories. The names of the repository/repositories and accession number(s) can be found in the article/**Supplementary Material**.

## Supplementary material

Supplementary Figure 1 Forest plots of the pooled **(A)** sensitivity, **(B)** specificity, and (c) SROC curve for CT-based radiomics with non-radiomics features in model in distinguishing between lung adenocarcinoma and lung squamous cell carcinoma.

Supplementary Figure 2 Forest plots of the pooled **(A)** sensitivity, **(B)** specificity, and **(C)** SROC curve for CT-based radiomics without non-radiomics features in model in distinguishing between lung adenocarcinoma and lung squamous cell carcinoma.

Supplementary Figure 3 Forest plots of the pooled **(A)** sensitivity, **(B)** specificity, and **(C)** SROC curve for CT-based radiomics models in distinguishing between lung adenocarcinoma and lung squamous cell carcinoma excluding two outlier studies.

Supplementary Figure 4 Forest plots of the pooled **(A)** sensitivity, **(B)** specificity, and **(C)** SROC curve for PET-CT-based radiomics models in distinguishing between lung adenocarcinoma and lung squamous cell carcinoma excluding one outlier study.

Supplementary Figure 5 Sensitivity analysis of the included studies developing radiomics models in distinguishing between lung adenocarcinoma and lung squamous cell carcinoma utilizing PET images: **(A)** goodness of fit, **(B)** bivariate normality, **(C)** influence analysis, and **(D)** outlier detection.

Supplementary Figure 6 Sensitivity analysis of the included studies developing radiomics models in distinguishing between lung adenocarcinoma and lung squamous cell carcinoma utilizing MRI images: **(A)** goodness of fit, **(B)** bivariate normality, **(C)** influence analysis, and **(D)** outlier detection.
