# Supplementary figures and images for: Radiomics in distinguishing between lung adenocarcinoma and lung squamous cell carcinoma: a systematic review and meta-analysis
Source: Front Oncol. 2024 Sep 24;14:1381217. doi: 10.3389/fonc.2024.1381217 (PMC11458374; doi:10.3389/fonc.2024.1381217)

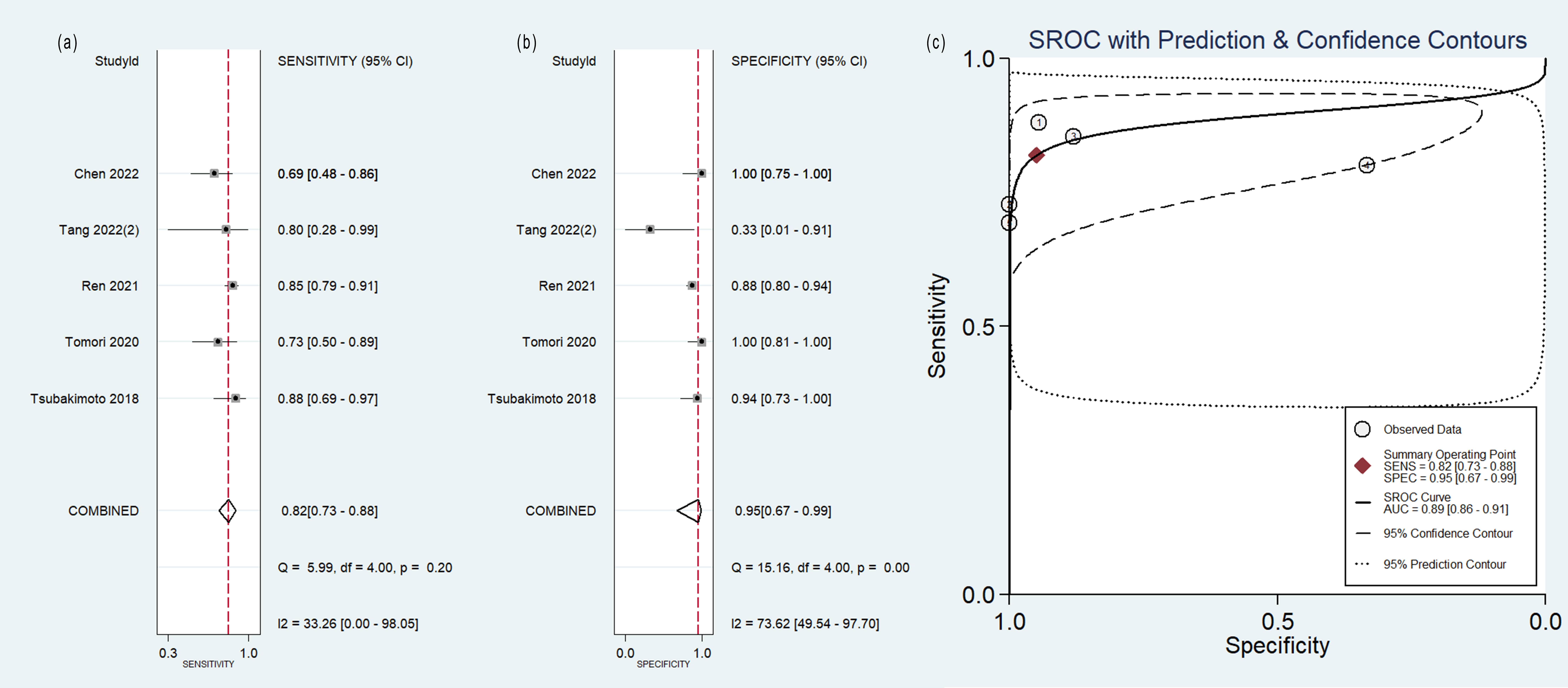

Supplement: Supplementary Figure 1 — Forest plots of the pooled (A) sensitivity, (B) specificity, and (c) SROC curve for CT-based radiomics with non-radiomics features in model in distinguishing between lung adenocarcinoma and lung squamous cell carcinoma. [file Image1.jpeg]

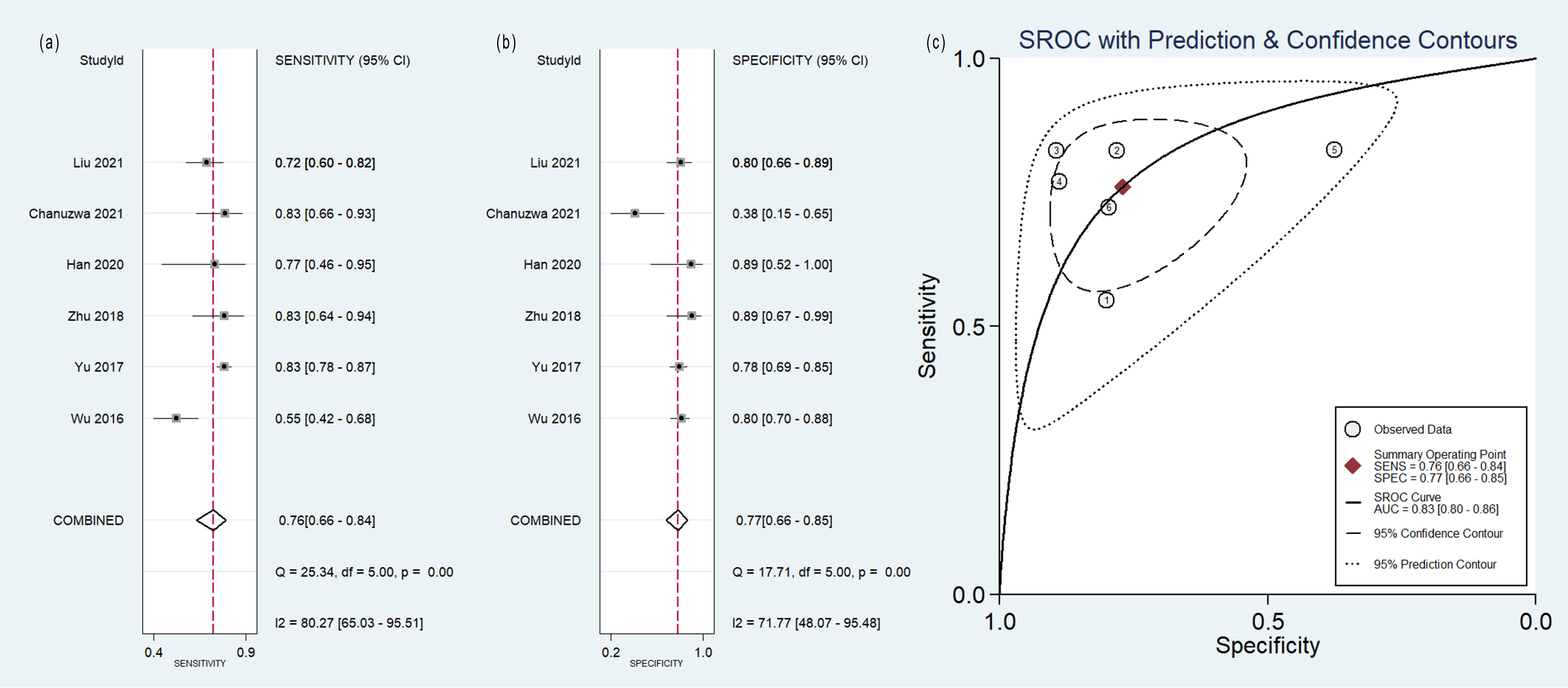

Supplement: Supplementary Figure 2 — Forest plots of the pooled (A) sensitivity, (B) specificity, and (C) SROC curve for CT-based radiomics without non-radiomics features in model in distinguishing between lung adenocarcinoma and lung squamous cell carcinoma. [file Image2.jpeg]

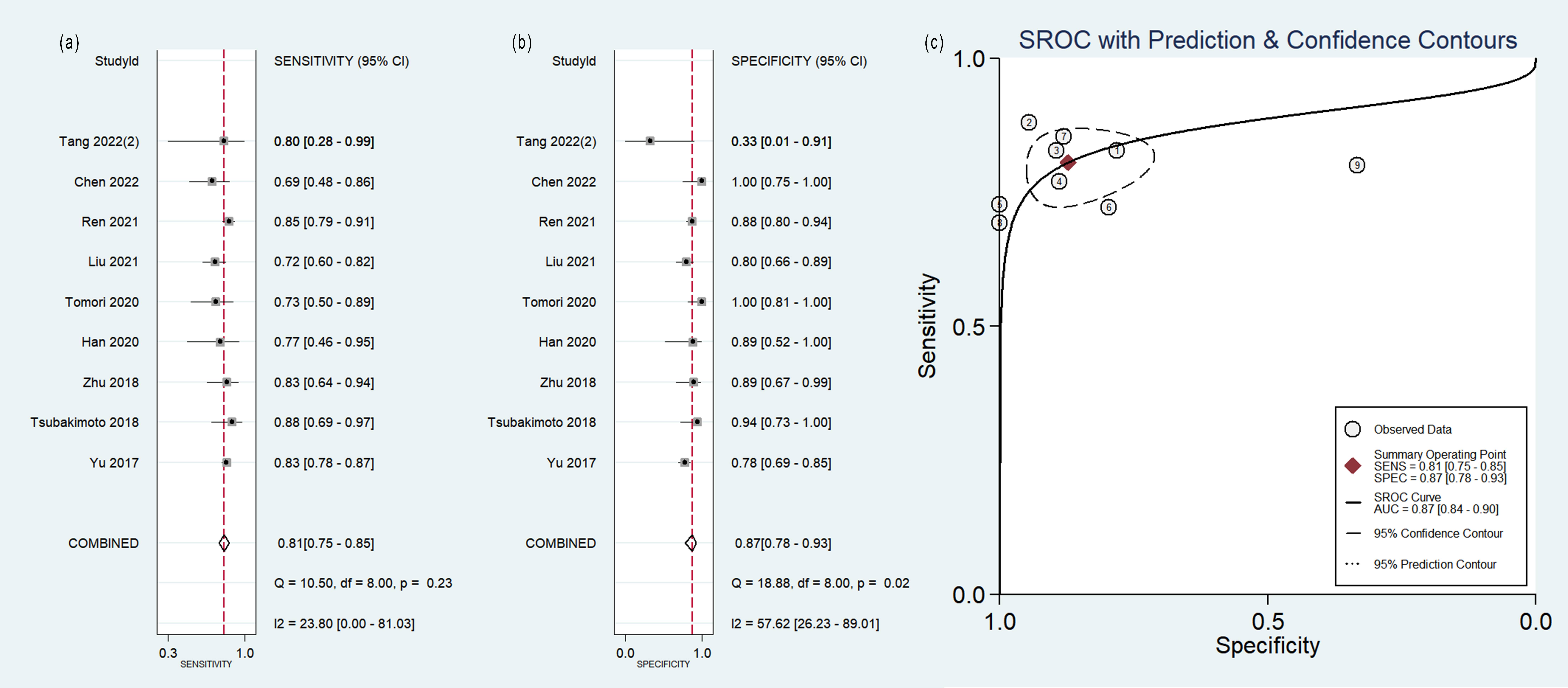

Supplement: Supplementary Figure 3 — Forest plots of the pooled (A) sensitivity, (B) specificity, and (C) SROC curve for CT-based radiomics models in distinguishing between lung adenocarcinoma and lung squamous cell carcinoma excluding two outlier studies. [file Image3.jpeg]

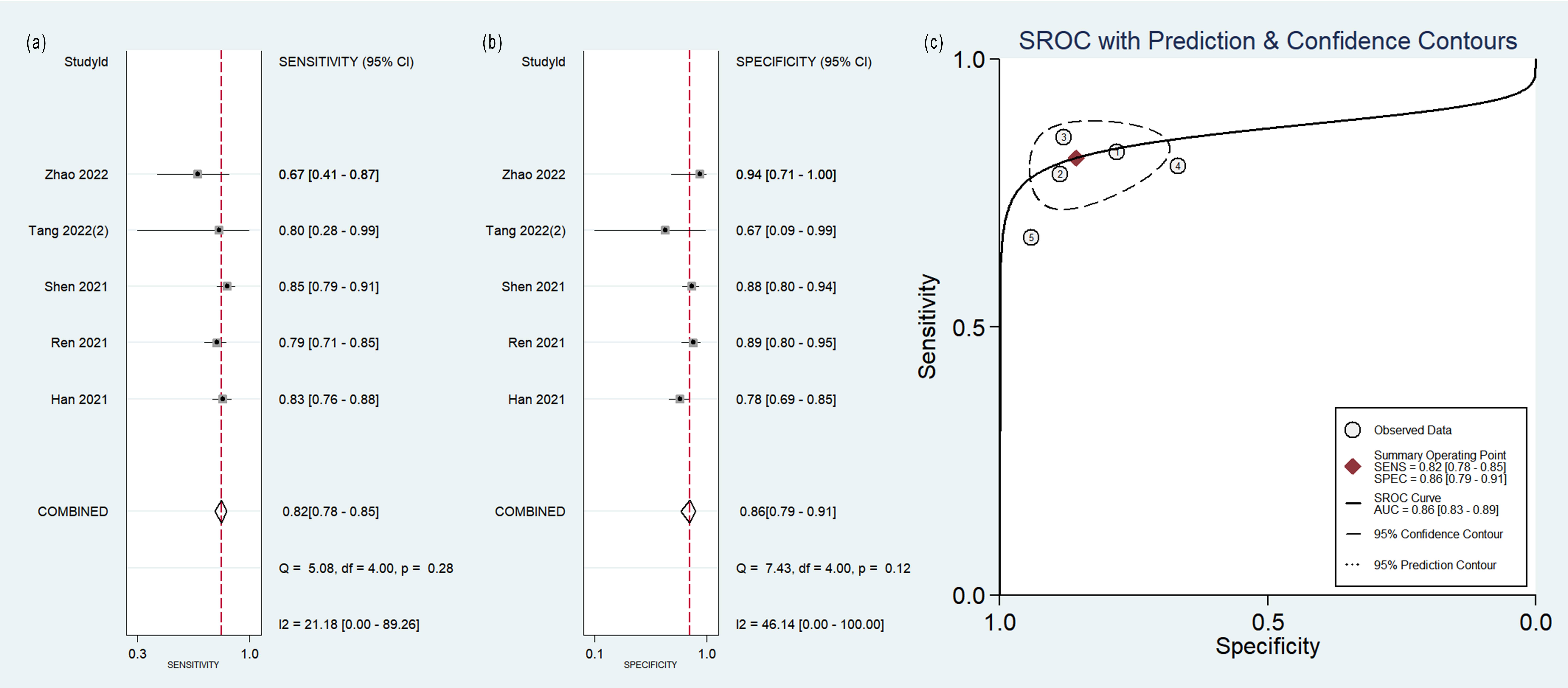

Supplement: Supplementary Figure 4 — Forest plots of the pooled (A) sensitivity, (B) specificity, and (C) SROC curve for PET-CT-based radiomics models in distinguishing between lung adenocarcinoma and lung squamous cell carcinoma excluding one outlier study. [file Image4.jpeg]

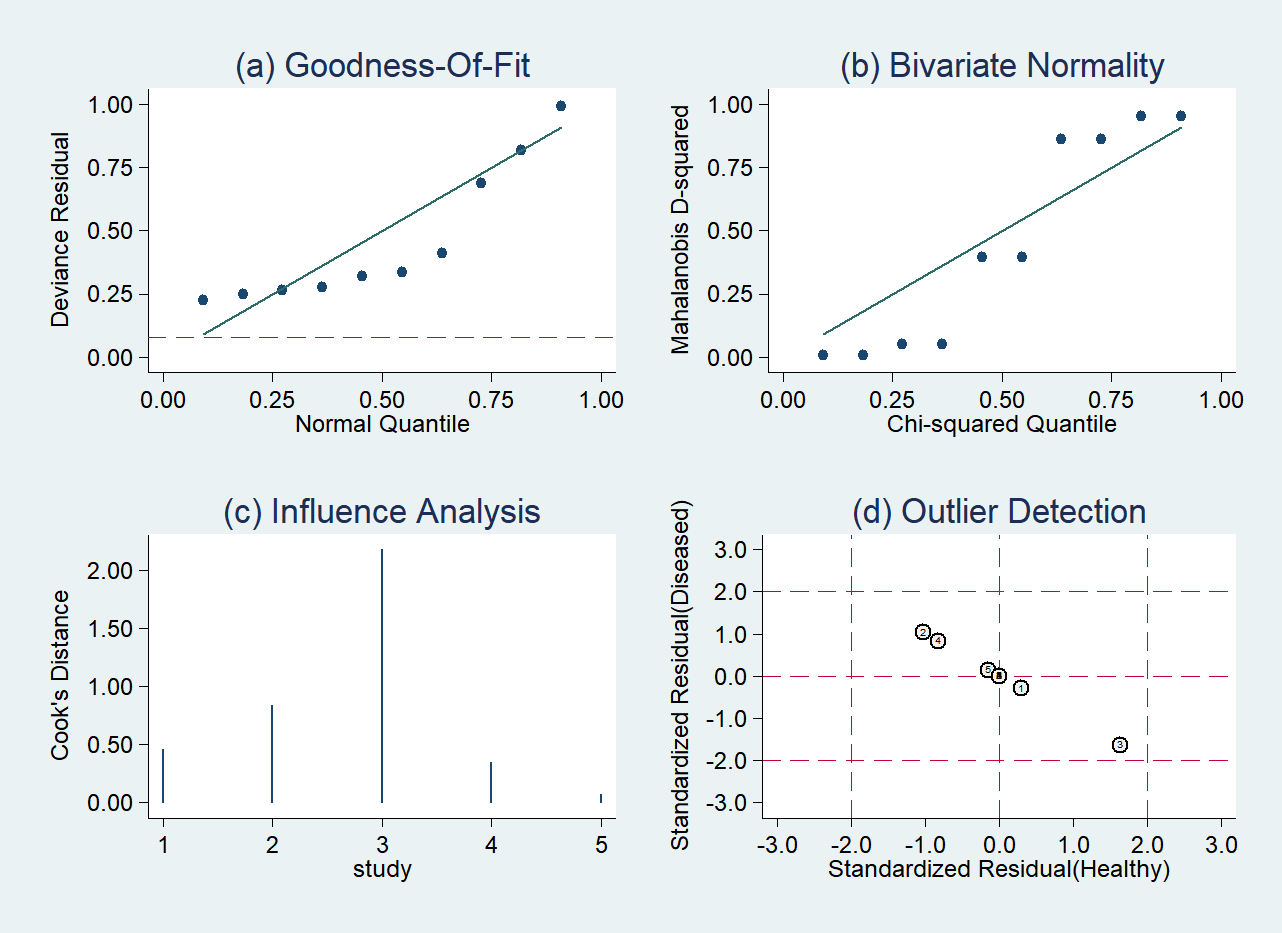

Supplement: Supplementary Figure 5 — Sensitivity analysis of the included studies developing radiomics models in distinguishing between lung adenocarcinoma and lung squamous cell carcinoma utilizing PET images: (A) goodness of fit, (B) bivariate normality, (C) influence analysis, and (D) outlier detection. [file Image5.tif]

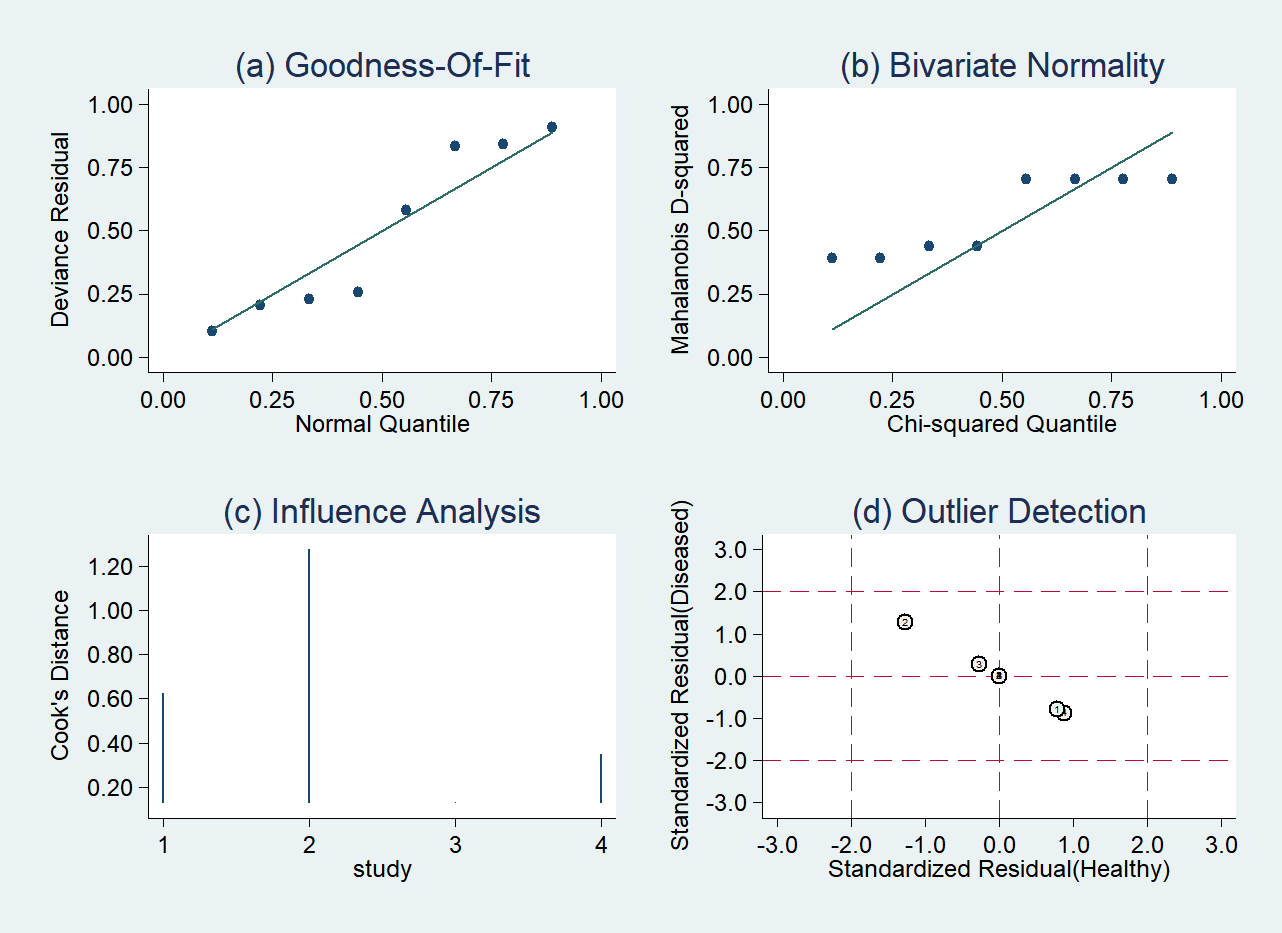

Supplement: Supplementary Figure 6 — Sensitivity analysis of the included studies developing radiomics models in distinguishing between lung adenocarcinoma and lung squamous cell carcinoma utilizing MRI images: (A) goodness of fit, (B) bivariate normality, (C) influence analysis, and (D) outlier detection. [file Image6.tif]
