# Supplementary material for: Radiomics in distinguishing between lung adenocarcinoma and lung squamous cell carcinoma: a systematic review and meta-analysis
Source: Front Oncol. 2024 Sep 24;14:1381217. doi: 10.3389/fonc.2024.1381217 (PMC11458374; doi:10.3389/fonc.2024.1381217)
Supplement: Supplementary file 8 [file Table2.docx]

Table S2 Search Strategy

| PubMed | | |
| --- | --- | --- |
| #1 | lung[tiab] OR pulmonary[tiab] OR lung[mesh] OR Carcinoma, Non-Small-Cell Lung[MeSH Terms] OR lung neoplasms[MeSH Terms] OR NSCLC[tiab] | 1,329,926 |
| #2 | Adenocarcinoma[tiab] OR adenocarcinomas[tiab] OR Adenocarcinoma[MeSH Terms] OR Adenocarcinoma of Lung[MeSH Terms] | 514,170 |
| #3 | squamous[tiab] OR Carcinoma, Squamous Cell[MeSH Terms] | 220,865 |
| #4 | Radiomic[tw] OR radiomics[tw] OR Quantitative[tw] OR first order[tw] OR histogram[tw] OR second order[tw] OR texture[tw] OR textural[tw] OR higher order[tw] OR wavelet[tw] | 971,921 |
| #5 | #1 AND #2 AND #3 AND #4 | 794 |
| Embase | | |
| #1 | 'lung'/exp | 429,628 |
| #2 | 'lung tumor'/exp | 549,455 |
| #3 | 'non small cell lung cancer'/exp | 210,775 |
| #4 | lung:ti,ab,kw OR pulmonary:ti,ab,kw OR nsclc:ti,ab,kw | 1,711,285 |
| #5 | #1 OR #2 OR #3 OR #4 | 1,925,683 |
| #6 | 'lung adenocarcinoma'/exp | 54,712 |
| #7 | 'adenocarcinoma'/exp | 348,467 |
| #8 | adenocarcinomas:ti,ab,kw OR adenocarcinoma:ti,ab,kw | 273,963 |
| #9 | #6 OR #7 OR #8 | 421,470 |
| #10 | 'squamous cell lung carcinoma'/exp | 16,435 |
| #11 | squamous:ti,ab,kw | 230,050 |
| #12 | radiomic:ti,ab,kw OR radiomics:ti,ab,kw OR quantitative:ti,ab,kw OR 'first order':ti,ab,kw OR histogram:ti,ab,kw OR 'second order':ti,ab,kw OR texture:ti,ab,kw OR textural:ti,ab,kw OR 'higher order':ti,ab,kw OR wavelet:ti,ab,kw | 1,164,178 |
| #13 | #5 AND #9 AND (#10 OR #11) AND #12 | 1,359 |
| Web of Science Core collection | | |
|  | (Topic: (lung OR pulmonary OR NSCLC) AND (Adenocarcinoma OR adenocarcinomas) AND squamous) AND  (Topic: Radiomic OR radiomics OR Quantitative OR first order OR histogram OR second order OR texture OR textural OR higher order OR wavelet) | 883 |

TableS3 The answers to nine signaling questions in four domains of improved QUADAS-2 of included studies

| StudyID | Patient selection | | | Index test | | | | Reference standard | Flow and timing |
| --- | --- | --- | --- | --- | --- | --- | --- | --- | --- |
|  | was a consecutive or random sample of patients enrolled? | was a case–control design avoided? | did the study avoid inappropriate exclusions? | were the imaging acquisition protocol, image processing approach described in detail? | were the segmentation method(s) described in detail? | were the feature  extraction software described in detail? | was the validation independent (i. e.  external)? | Is the reference standard likely to correctly classify the target condition? | Was there an appropriate  interval between index test and reference standard? |
| Basu 2011 | unclear | unclear | unclear | no | yes | yes | no | yes | unclear |
| Wu 2016 | yes | yes | yes | yes | yes | yes | yes | yes | yes |
| Yu 2017 | unclear | unclear | yes | yes | yes | no | no | yes | unclear |
| Haga 2018 | yes | yes | yes | yes | yes | yes | yes | yes | yes |
| Sandino 2018 | yes | yes | yes | yes | yes | no | no | yes | yes |
| Tsubakimoto 2018 | yes | yes | no | yes | yes | yes | no | yes | yes |
| Zhu 2018 | yes | yes | yes | yes | yes | yes | yes | yes | yes |
| Bashir 2019 | yes | yes | yes | yes | yes | yes | yes | yes | yes |
| Digumarthy 2019 | yes | yes | yes | yes | yes | yes | no | yes | yes |
| E 2019 | yes | yes | yes | yes | yes | yes | no | yes | yes |
| Liu 2019 | no | no | no | yes | yes | yes | no | yes | yes |
| Yamada 2019 | no | no | yes | yes | no | no | no | yes | yes |
| Alvarez-Jimenez 2020 | yes | yes | yes | yes | yes | no | no | yes | yes |
| Brunese 2020 | no | no | yes | yes | no | no | no | yes | yes |
| Han 2020 | yes | yes | yes | yes | yes | yes | yes | yes | yes |
| Tomori 2020 | yes | yes | no | yes | yes | yes | no | yes | yes |
| Vuong 2020 | yes | yes | yes | yes | yes | yes | yes | yes | yes |
| Chanuzwa 2021 | yes | yes | no | no | yes | yes | yes | yes | yes |
| Li 2021 | yes | yes | yes | yes | yes | yes | no | yes | yes |
| Liu 2021 | yes | yes | no | yes | yes | yes | yes | yes | yes |
| Marentakis 2021 | no | no | yes | yes | no | no | yes | yes | yes |
| Chen 2022 | yes | yes | no | yes | yes | yes | yes | yes | yes |
| Tang 2022(1) | yes | yes | yes | yes | yes | yes | yes | yes | yes |
| Chen 2023 | yes | yes | yes | yes | no | yes | yes | yes | yes |
| Song 2023 | yes | yes | yes | yes | yes | yes | yes | yes | yes |
| Ha 2014 | yes | yes | yes | yes | yes | yes | no | yes | yes |
| Ma 2018 | yes | yes | yes | yes | yes | yes | no | yes | yes |
| Hyun 2019 | yes | yes | yes | yes | no | yes | yes | yes | yes |
| Sha 2019 | yes | yes | yes | yes | yes | yes | yes | yes | yes |
| Ayyildiz 2020 | yes | yes | yes | no | yes | yes | no | yes | unclear |
| Han 2021 | yes | yes | yes | yes | yes | yes | yes | yes | yes |
| Ji 2021 | yes | yes | yes | yes | yes | yes | yes | yes | yes |
| Ren 2021 | yes | yes | yes | yes | yes | yes | yes | yes | yes |
| Shen 2021 | yes | yes | yes | yes | yes | yes | no | yes | unclear |
| Zhou 2021 | yes | yes | yes | yes | yes | yes | yes | yes | yes |
| Zhao 2022 | yes | yes | yes | yes | yes | yes | yes | yes | yes |
| Tang 2020 | yes | yes | yes | yes | yes | yes | yes | yes | yes |
| Yang 2023 | yes | yes | no | yes | yes | yes | yes | yes | yes |
| Bebas 2021 | no | no | yes | no | no | yes | no | yes | unclear |
| Tang 2022(2) | yes | yes | yes | yes | yes | yes | yes | yes | yes |
| Inter-rater  agreement (100%) | 91.6 | 91.6 | 92.0 | 86.3 | 100.0 | 100.0 | 95.0 | 100.0 | 100.0 |

Note: The signaling questions for the 4 key domains of QUADAS-2 were modified to suit our study. In the domain of patient selection, we used the original signaling questions. In the domain of index test, the original signaling questions “were the index test results interpreted without knowledge of the results of the reference standard?” and “if a threshold was used, was it prespecified?” were removed. Because a threshold might not be predetermined for radiomics features, and reference standard results would have limited influence on the interpretation of the results. Three signaling questions specific to radiomics methodology and related to the risk of bias of radiomics workflow were added to the domain of index test, namely “were the imaging acquisition protocol, image processing approach described in detail?”, “were the segmentation method(s) described in detail?” and “were the feature extraction software described in detail?”. In the domain of reference standard, the original signaling question “were the reference standard results interpreted without knowledge of the results of the index test?” was not used due to the results interpretation by reference standard often preceded before the results interpretation by the radiomics model. In the domain of follow and timing, two original signaling questions “did all patients receive a reference standard?” and “did all patients receive the same reference standard?” were not used because all patients had to be assessed with adequate standards prior to beginning the radiomics flow. The original signaling question “were all patients included in the analysis?” were not used because not all the patients were included in the analysis according to the nature of the radiomics workflow.

Table S4 True positive, False negative, false positive and true negative of 21 studies included in meta-analysis

| Study ID | Imaging modality | TP | FN | FP | TN |
| --- | --- | --- | --- | --- | --- |
| Wu 2016 | CT | 34 | 28 | 18 | 72 |
| Yu 2017 | CT | 268 | 56 | 24 | 86 |
| Tsubakimoto 2018 | CT | 22 | 3 | 1 | 17 |
| Zhu 2018 | CT | 24 | 5 | 2 | 17 |
| Han 2020 | CT | 10 | 3 | 1 | 8 |
| Tomori 2020 | CT | 16 | 6 | 0 | 18 |
| Chanuzwa 2021 | CT | 29 | 6 | 10 | 6 |
| Liu 2021 | CT | 52 | 20 | 11 | 43 |
| Ren 2021 | CT | 128 | 22 | 12 | 88 |
|  | PET | 126 | 14 | 30 | 50 |
|  | PET-CT | 110 | 30 | 9 | 71 |
| Chen 2022 | CT | 18 | 8 | 0 | 13 |
| Tang 2022(2) | CT | 4 | 1 | 2 | 1 |
|  | PET | 4 | 1 | 1 | 2 |
|  | PET-CT | 4 | 1 | 1 | 2 |
|  | MRI | 3 | 2 | 1 | 2 |
|  | PET-MRI | 4 | 1 | 1 | 2 |
| Hyun 2019 | PET | 157 | 53 | 38 | 148 |
| Sha 2019 | PET | 16 | 0 | 3 | 7 |
| Ayyildiz 2020 | PET | 18 | 21 | 3 | 51 |
| Ma 2018 | PET-CT | 124 | 1 | 7 | 167 |
| Han 2021 | PET-CT | 143 | 30 | 24 | 86 |
| Shen 2021 | PET-CT | 128 | 22 | 12 | 88 |
| Zhao 2022 | PET-CT | 12 | 6 | 1 | 16 |
| Tang 2020 | MRI | 22 | 8 | 2 | 16 |
| Bebas 2021 | MRI | 19 | 5 | 6 | 14 |
| Yang 2023 | MRI | 9 | 5 | 1 | 8 |

TP: True positive, FN: False negative, FP: false positive, TN: true negative.
